# 

**Published:** 1903-12-15

**Authors:** 


					﻿THE
DENTAL REGISTER
______~_EDITED BY	X
"Ti rWHLVltXE s. HOFF, D*. D.S.,/
cuPuEONGt-N'‘"J	7
' Jr	ANN ARBOR, MICH.
— ■	—L______I_____
Vol. LVH. DECEMBER 15, 1903.	No-ia/
PRICE, ONE DOLLAR A YEAR IN ADVANCE.
PUBLISHED MONTHLY BY
SAMUEL A. CROCKER & CO.,
THE OHIO DENTAL AND SURGICAL DEPOT.
33 to 30 W. £>tlx St., Cinciiinnti, O.
Entered at the Postoffice at Cincinnati, 0., as second-class mail matter.
				

